# Differential Effects of Isoxazole-9 on Neural Stem/Progenitor Cells, Oligodendrocyte Precursor Cells, and Endothelial Progenitor Cells

**DOI:** 10.1371/journal.pone.0138724

**Published:** 2015-09-25

**Authors:** Seong-Ho Koh, Anna C. Liang, Yoko Takahashi, Takakuni Maki, Akihiro Shindo, Noriko Osumi, Jing Zhao, Hong Lin, Julie C. Holder, Tsu Tshen Chuang, John D. McNeish, Ken Arai, Eng H. Lo

**Affiliations:** 1 Neuroprotection Research Laboratory, Departments of Radiology and Neurology, Massachusetts General Hospital and Harvard Medical School, Charlestown, Massachusetts, United States of America; 2 Department of Neurology, Hanyang University College of Medicine, Seoul, Korea; 3 Department of Developmental Neuroscience, United Centers for Advanced Research and Translational Medicine, Tohoku University Graduate School of Medicine, Sendai, Japan; 4 Regenerative Medicine Discovery Performance Unit, GlaxoSmithKline, Cambridge, Massachusetts, United States of America; University of South Florida, UNITED STATES

## Abstract

Adult mammalian brain can be plastic after injury and disease. Therefore, boosting endogenous repair mechanisms would be a useful therapeutic approach for neurological disorders. Isoxazole-9 (Isx-9) has been reported to enhance neurogenesis from neural stem/progenitor cells (NSPCs). However, the effects of Isx-9 on other types of progenitor/precursor cells remain mostly unknown. In this study, we investigated the effects of Isx-9 on the three major populations of progenitor/precursor cells in brain: NSPCs, oligodendrocyte precursor cells (OPCs), and endothelial progenitor cells (EPCs). Cultured primary NSPCs, OPCs, or EPCs were treated with various concentrations of Isx-9 (6.25, 12.5, 25, 50 μM), and their cell numbers were counted in a blinded manner. Isx-9 slightly increased the number of NSPCs and effectively induced neuronal differentiation of NSPCs. However, Isx-9 significantly decreased OPC number in a concentration-dependent manner, suggesting cytotoxicity. Isx-9 did not affect EPC cell number. But in a matrigel assay of angiogenesis, Isx-9 significantly inhibited tube formation in outgrowth endothelial cells derived from EPCs. This potential anti-tube-formation effect of Isx-9 was confirmed in a brain endothelial cell line. Taken together, our data suggest that mechanisms and targets for promoting stem/progenitor cells in the central nervous system may significantly differ between cell types.

## Introduction

Neuronal loss is one of the most important and common features in numerous intractable neurological diseases such as stroke, central nervous system (CNS) trauma, Alzheimer’s disease, Parkinson’s disease, and amyotrophic lateral sclerosis. Theoretically, if endogenous neurogenesis can be enhanced, then one might pursue the therapeutic possibility of replacing lost neurons and boosting functional recovery [1–3]. Although this remains a compelling idea and goal, to date, there have been no clinically validated approaches for enhancing neurogenesis and neural replacement.

Recently, it has been suggested that CNS recovery therapies should not be “neurocentric” [4]. Proper neural function requires not only viable neurons, but also integrated crosstalk between all cell types from neural, glial and vascular compartments [1,4,5]. Neuroprotection and neurorepair cannot exist in isolation. Clinically effective therapies must rescue all cell types in the recovering CNS. Hence, any therapeutic approach that seeks to promote neurogenesis should also consider effects on gliogenesis and angiogenesis [1,4,5].

Isoxazole 9 (Isx-9) is a synthetic small molecule that promotes adult neurogenesis [6,7]. It has been shown that Isx-9 can trigger neuronal differentiation of adult neural stem/precursor cells (NSPCs) through Ca^2+^ influx which activates phosphorylated CaMK and mediated nuclear export of the MEF2 regulator HDAC5, thus de-repressing neuronal genes and allowing differentiation. In this study, we ask whether Isx-9 may also affect other precursor cell populations in the CNS. Therefore, we conducted in vitro proof-of-concept studies wherein Isx-9 was administered to primary rat NSPCs, primary rat oligodendrocyte precursor cells (OPCs), and primary mouse endothelial progenitor cells (EPCs), which together may comprise a representation of the major progenitor/precursor pools relevant for recovering neural, glial and vascular elements in damaged or diseased brain.

## Material and Methods

All experiments were performed following an institutionally approved protocol in accordance with the National Institutes of Health Guide for the Care and Use of Laboratory Animals, and NIH Guidelines on Ethical Animal Use and Rigor. All experiments and procedures were conducted following a Massachusetts General Hospital (MGH) IACUC-approved protocol. The MGH Subcommittee on Research Animal Care (institutional IACUC) reviewed and approved all experiments conducted here. These IACUC approval protocols and studies were all in compliance with the MGH ethics review procedure, and conducted in compliance with GlaxoSmithKline policy on care, welfare and treatment laboratory animals. All experimental animals were sacrificed by decapitation under deep isoflurane anesthesia, according to IACUC-approved protocols. All procedures and analyses were performed following standard requirements for experimental design including allocation concealment, randomization, blinding and statistical powering (www.ninds.nih.gov/funding/transparency_in_reporting_guidance.pdf). 

### Cultured Rat Neural Stem/Progenitor Cells

Cultured rat NSPCs were prepared according to our previous report [8,9]. Briefly, embryonic brain tissue was dissected from the cortex, lateral ganglionic eminence, and ventral midbrain from embryonic day 12–13 Sprague-Dawley rats. After mechanical trituration, 20,000 cells/cm^2^ were plated in culture dishes precoated with poly-L-ornithine (PO)/fibronectin (FN) in phosphate-buffered saline (PBS) and cultured in N2 medium [DMEM/F12, 25 mg/L insulin, 100 mg/L transferrin, 30 nM selenite, 0.6 mM putrescine, 20 nM progesterone, 2 mM L-glutamine, 8.6 mM D(+) glucose, 20 mM NaHCO_3_, 1% penicillin/streptomycin] supplemented with basic fibroblast growth factor (bFGF, 20 ng/mL; R&D Systems) for 4–6 days as a monolayer on the adherent surface. To obtain a uniform population of NSPCs, clusters of cells, which represent proliferating cells because of the presence of a mitogen (bFGF), were passaged by dissociating the clusters into single cells and plating these cells in freshly PO/FN-coated flasks (NUNC, Roskilde, Denmark) [8,9]. All data in this study were obtained from passaged cultures grown on adherent surfaces. Cultures were maintained at 37°C in a 5% CO_2_ incubator. Media were changed every other day and mitogens were added daily.

### Cultured Rat Oligodendrocyte Precursor Cells

Cultured rat OPCs were prepared according to our previous report [10]. Briefly, primary mixed glial cultures were prepared from postnatal day 0–2 Sprague-Dawley rat pups by mechanical dissociation. Isolated cortices were minced and digested for 15 min in 5 mL Hank's balanced salt solution (Gibco) containing 190 uL DNase and 50 uL 2.5% Trypsin. Media were changed every other day with 20% Fetal Bovine Serum (Gibco) in Dulbecco's modified eagle medium (DMEM) (Gibco). Ten to eleven day old cultures were shaken for 1 hour to dissociate microglial and then shaken overnight to detach oligodendrocyte progenitors from the astrocyte monolayer. To minimize contamination by microglial cells, the detached cell suspension was incubated for 1 hour in 100 mm dishes. The non-adherent cells were resuspended in Neurobasal medium (Gibco) containing B27 supplement, PDGF (10 ng/mL) and FGF (10 ng/mL) and 1mL of cell suspension was seeded in poly-D-ornithine (5mg/mL) coated 12 well plates. Media were changed every 2–3 days.

### Cultured Mouse Endothelial Progenitor Cells

Cultured rat EPCs were prepared according to our previous report [11,12]. Briefly, for each independent experiment, spleens from 10 weeks old CD-1 mice were obtained and kept in PBS solution. Under the hood, the spleens were mechanically minced, placed at 37°C for 15 min with lysis buffer [4% BSA and 1mM EDTA in PBS] and run through a 40 μm nylon membrane to obtain cell suspension. Subsequently, mononuclear cells (MNCs) were obtained by density gradient centrifugation using Ficoll-Paque Plus (Amersham Biosciences Corp, Piscataway, NJ, USA). Isolated MNCs were shortly washed with red blood cell lysis buffer [155 mM NH4Cl, 10 mM NaHCO3, and EDTA in H2O] and gently washed twice with complete growth media EGM-2MV (Lonza, Hopkinton, MA, USA) consisting of endothelial basal medium-2 (EBM-2), 5% fetal bovine serum (FBS), hEGF, VEGF, hFGF-B, IGF-1, ascorbic acid, and heparin. MNCs were finally resuspended in EGM-2MV, seeded on collagen-coated twelve well plates (Becton Dickinson Labware BD, Franklin Lakes, NJ, USA), and incubated in a 5% CO_2_ incubator at 37°C. Under daily observation, first medium change was done 3 days after seeding of MNCs and medium was changed every 2 days. Early EPCs were used 5–6 days after seeding of MNCs for the evaluation of cell proliferation/survival and outgrowth EPCs were used 1–1.5 months after seeding of MNCs for tube formation assay. To confirm effects on angiogenesis, matrigel experiments were repeated using human brain endothelial cells (CSC, Kirkland, WA) [13].

### Isx-9 Treatment

Isx-9 was purchased from Tocris (Tocris Bioscience, Boston, MA, USA) and dissolved in DMSO. Cells (NSPCs, OPCs, early EPCs, or outgrowth EPCs) were treated with different concentrations of Isx-9 (0, 6.25, 12.5, 25, or 50 μM), and 2 days later, cell numbers were counted in a blinded manner to evaluate the effects of Isx-9 on the proliferation/survival of four types of progenitor/precursor cells. NSPC proliferation/survival was also assessed by WST reduction assay (Cell Counting Kit-8, Dojindo). WST assay is a sensitive colorimetric method to detect cell viability. NSPCs were incubated with 10% WST solution for 1 h at 37°C. Then the absorbance of the culture medium was measured with a microplate reader at a test wavelength of 450 nm and a reference wavelength of 630 nm.

For differentiation experiments for NSPCs, endpoints were measured at 5 days.

### Immunocytochemistry

To confirm the status/purity of primary cultured NSPCs, the cells were stained with nestin (1:100, Abcam, Cambridge, MA, USA) and Ki67 (1:100, Abcam) antibodies, which are well-known markers for NSPCs [14,15]. To assess the effects of Isx-9 on in vitro differentiation of NSPCs, the Isx-9-treated were stained for neuronal surface antigens with neurofilament (1:100, Abcam) and NeuN (1:100, Millipore Corporation, Billerica, MA, USA) antibodies, which are the most commonly used markers for differentiated neurons [16]. OPCs were stained for cell surface antigens with PDGF-R-a antibody (1:300) as previously described [17]. For EPCs, immunocytochemistry was performed 5–6 days after seeding of MNCs for early EPCs and 1–1.5 months for outgrowth EPCs. Direct fluorescent staining was used to detect lectin binding with fluorescein isothiocyanate (FITC)-labeled Ulex Europaeus Agglutin (UEA)-1 (Sigma, St Louis, MO, USA). At the same time, surface-antigen expression of markers including CD31 (1:100, BD Bioscience, NJ, USA), CD34 (1:100, Santa Cruz, Dallas, TX, USA), CD133 (1:100, Abcam, Cambridge, MA, USA), Flk-1/VEGFR2 (1:100, Santa Cruz, Dallas, TX, USA), and von Willebrand Factor (vWF) (Dakocytomation, Glostrup, Denmark) were used for the identification of EPCs. Procedures for immunocytochemistry were as previously described [11,12]. Briefly, cells were washed three times with PBS on ice. After washing with PBS, the cells were fixed in 4% paraformaldehyde for 10 min at room temperature. Then rinsed once with PBS-T (0.1% tween 20 in PBS). After the wash, the cells were blocked using 3% BSA in PBS-T for 1 hour, followed by overnight incubation with primary antibodies at 4°C. The cells were then washed three times for 5 min in PBS-T, and incubated for 1 hour with secondary antibody diluted 1:100 in 0.3% BSA in PBS-T. The cells were washed three times for 5 min using PBS-T, mounted with Vectashield containing DAPI, and visualized using fluorescent microscope (Nikon).

### Western Blot

To assess the effects of Isx-9 on in vitro differentiation of NSPCs, the levels of nestin, neurofilament, and NeuN in Isx-9-treated NSPCs were analyzed. Cells were washed twice in cold PBS and incubated in lysis buffer [50 mM Tris (pH 8.0), 150 mM NaCl, 0.02% sodium azide, 0.2% SDS, 100 μg/ml phenylmethylsulfonylfluoride (PMSF), 50 μl/ml aprotinin, 1% Igepal 630, 100 mM NaF, 0.5% sodium deoxycholate, 0.5 mM EDTA, and 0.1 mM EGTA] for 10 min on ice. The cell lysates were centrifuged at 10,000 g and the amounts of nestin, neurofilament, and NeuN in the cell lysate were determined. Protein concentrations of cell lysates were determined using a Bio-Rad protein assay kit. Samples containing equal amounts (20 μg) of protein were resolved by 10% sodium dodecyl sulfate-polyacrylamide gel electrophoresis (SDS-PAGE) and transferred to nitrocellulose membranes (Amersham Pharmacia Biotech, Buckinghamshire, UK). The membranes were blocked with 5% skim milk before incubation with specific primary antibodies against nestin (1:1000, Abcam), neurofilament (1:1000, Abcam), and NeuN (1:1000, Millipore). The membranes were washed with Tris-buffered saline containing 0.05% Tween-20 (TBST) and then processed using a horseradish peroxidase (HRP)-conjugated anti-rabbit or anti-mouse antibody (Amersham Pharmacia Biotech, Piscataway, NJ, USA) followed by enhanced chemiluminescence (ECL) detection (Amersham Pharmacia Biotech). The results from the Western blots were quantified with an image analyzer (Bio-Rad, Quantity One-4, 2,0).

### Matrigel Tube Formation Assay

The standard Matrigel method was used as a surrogate in vitro angiogenesis assay to assess the spontaneous formation of capillary-like structures of seeded vascular cells. Standard 15-well plates were coated with 12 μl of cold Matrigel and allowed to be solidified at 37°C for 30 min. Outgrowth EPCs or rat brain endothelial RBE.4 cells (2×10^5^ cells/ml) were seeded into the plates and incubated at 37°C for 18 hour with different concentrations of Isx-9. The degree of tube formation was determined by counting the number of tubes in five random fields from each well under a 10x objective.

### Statistical analysis

All data are expressed as mean ± SD from three or more independent experiments performed in triplicate. Statistical comparisons of variability and Western blot data between different treatment groups were performed by using ANOVA followed by post hoc Tukey-Kramer tests with SPSS (version 21.0, SPSS Inc., Chicago, IL, USA). P-values less than 0.05 were considered statistically significant.

## Results

### Effects of Isx-9 on NSPCs

**Fig 1:**
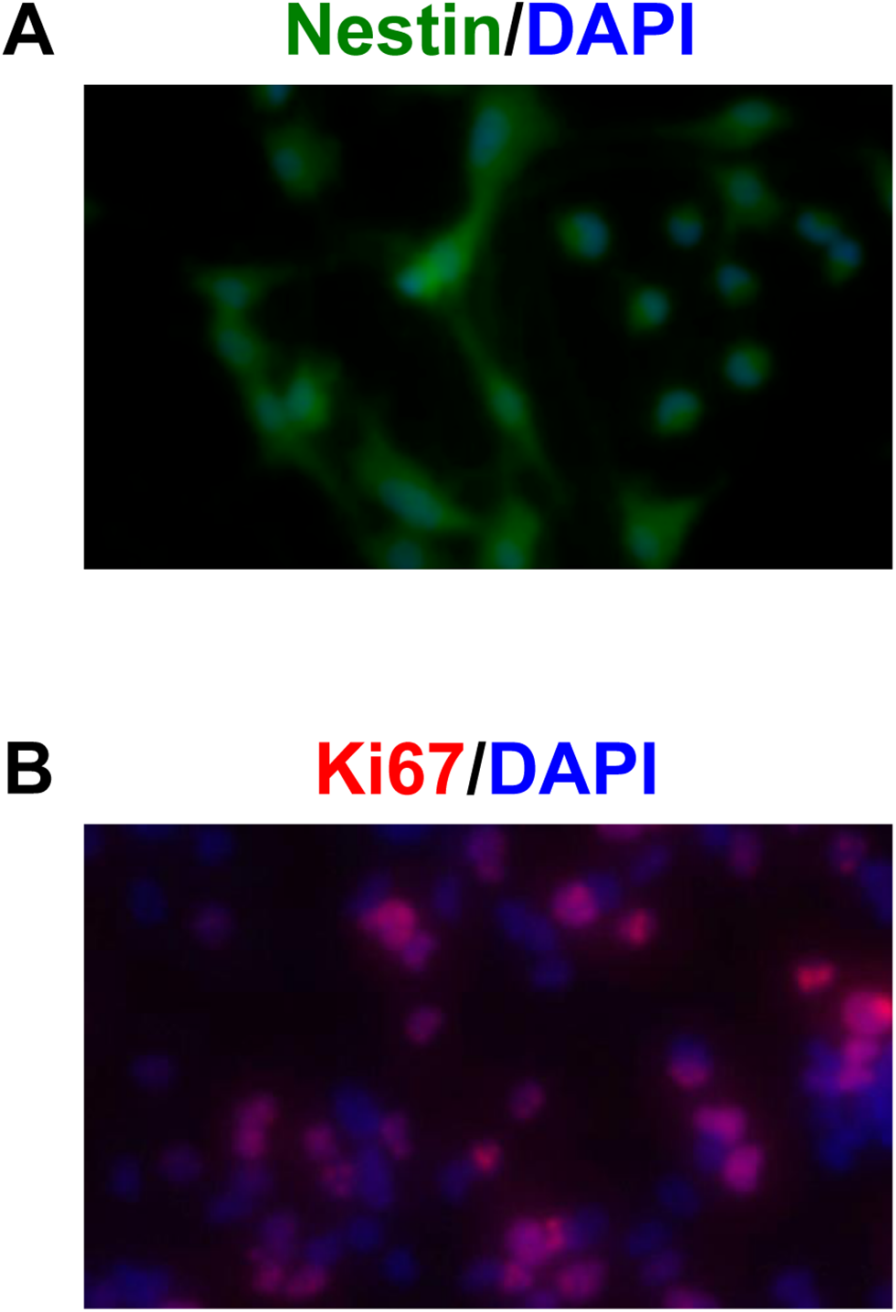
NSPC cultures. Immunohistochmistry confirmed that our NSPC cultures were positive for a NSPC marker nestin (A) and a proliferative marker Ki67 (B). Nuclei were stained by DAPI shown in blue.

**Fig 2:**
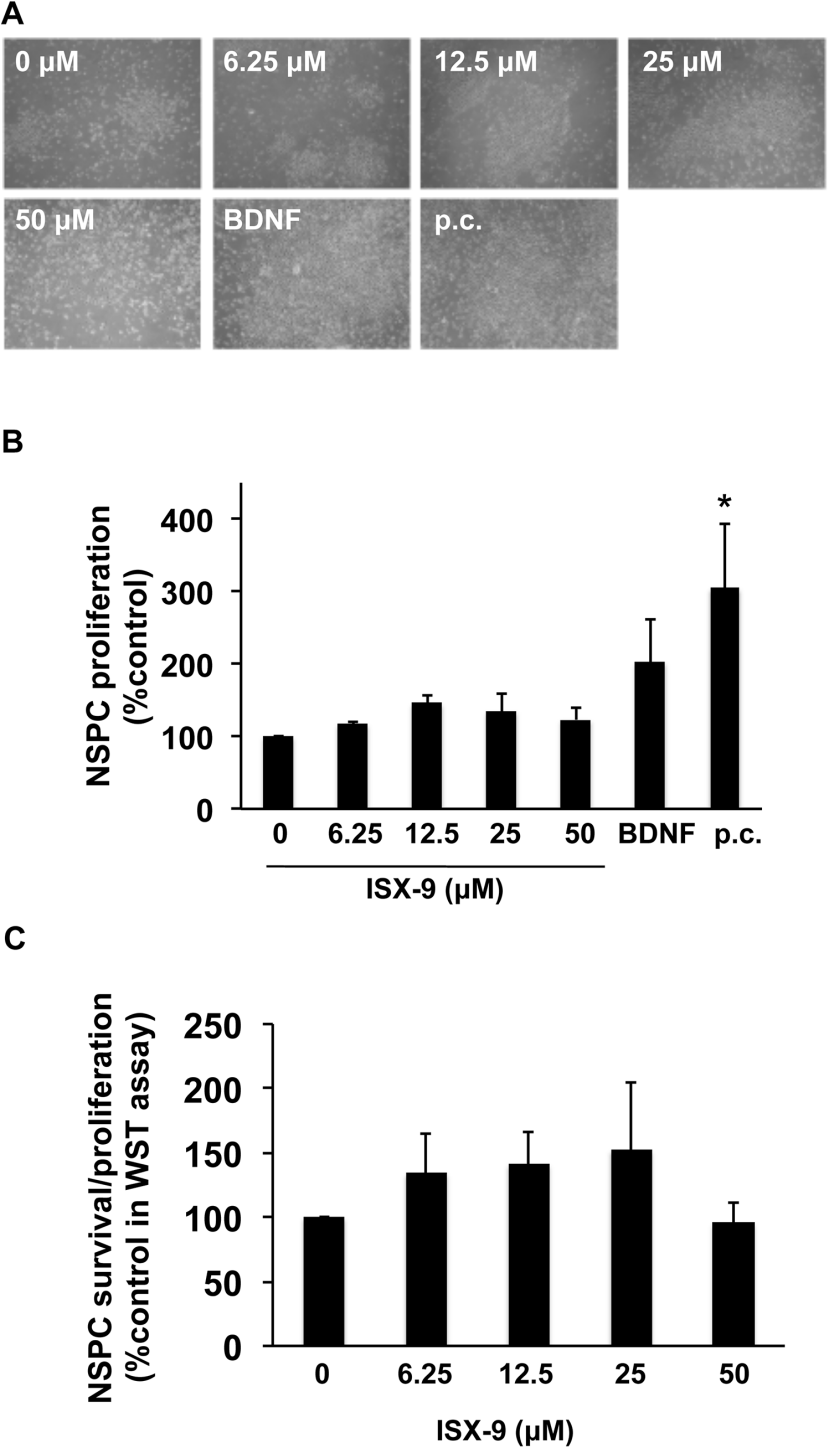
Isx-9 increased the number of NSPCs. NSPC cultures were treated with various concentrations of Isx-9, and two days later, their proliferation was evaluated by direct counting. Maintaining cells in the proliferative media was used as a positive control to proliferate NSPCs. (A) Representative images of NSPCs after Isx-9 treatment. (B) Isx-9 treatment slightly promoted the proliferation of NSPCs. The positive control indeed increased the number of NSPCs. p.c. indicates positive control. (C) WST assay shows similar responses to Isx-9 in NSPCs. Data are mean ± SD. *p<0.05 vs. control group.

As expected, NSPCs were positive for the standard marker nestin [14,15] and a proliferative marker Ki67 (Fig 1). As a positive control, NSPCs were treated with BDNF (10 ng/mL) or maintained in their original proliferation medium containing diverse trophic factors. Under the positive control conditions of proliferation medium, NSPCs were observed to proliferate (Fig 2A and 2B). When NSPCs were treated with Isx-9 (6.25–50 uM) for 2 days, cell counts were slightly increased (Fig 2A and 2B). These cell counting data were supported by WST mitochondrial viability assays (Fig 2C).

**Fig 3:**
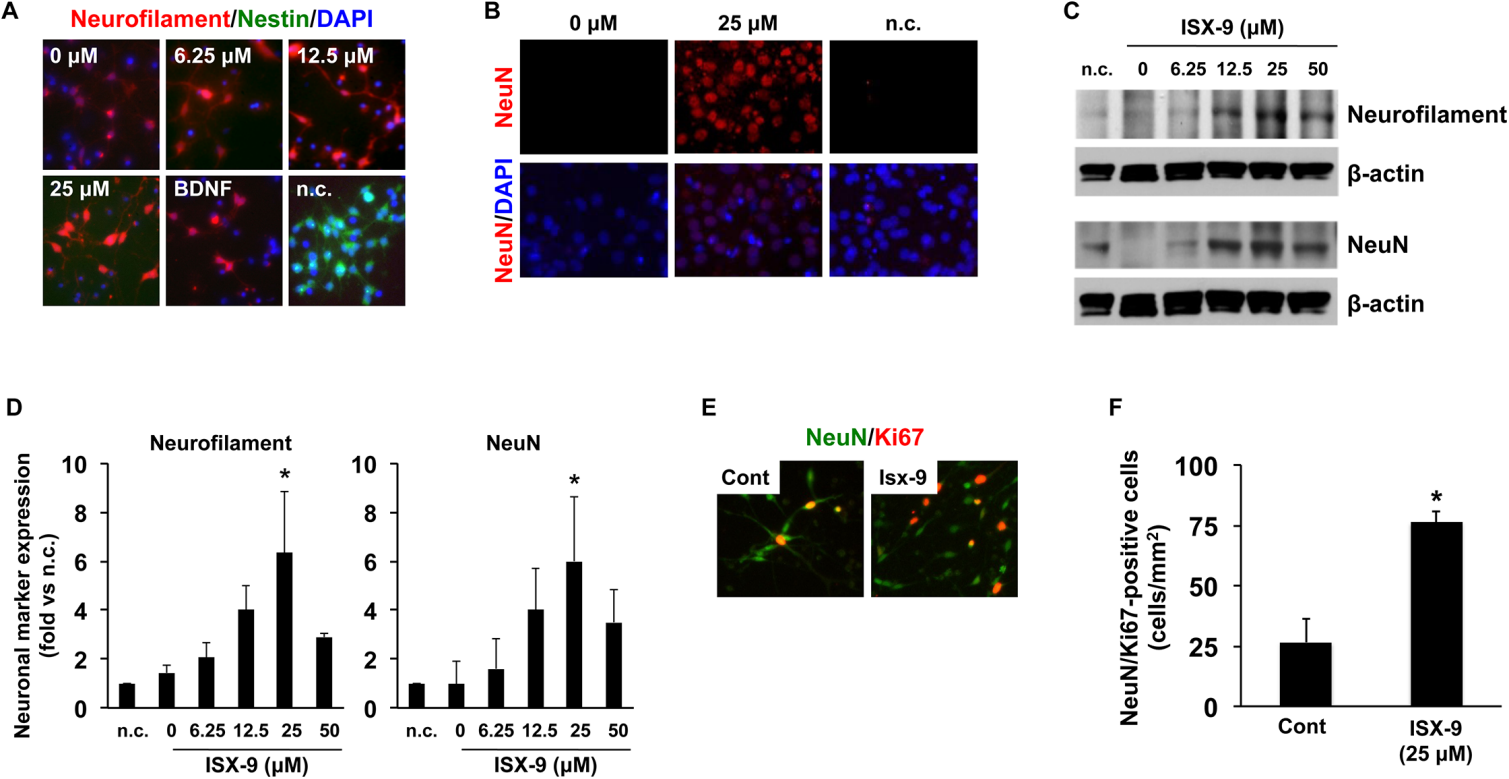
Isx-9 promoted the differentiation of NSPCs. NSPC cultures were treated with various concentrations of Isx-9, and five days later, their differentiation into neurons was evaluated by immunostaining. As BDNF is well-known to differentiate NSPCs into neurons, BDNF treatment (10 ng/mL) was used to validate our experimental system. Maintaining cells in the proliferative media were used as a negative control for NSPC differentiation. (A-B) Immunostaining using neuron markers neurofilament and NeuN showed that Isx-9 promoted the differentiation into neurons. (C-D) Western blotting confirmed that expressions of neurofilament and NeuN were increased by Isx-9 treatment. n.c. indicates negative control. (E) Representative images of Ki67-NeuN double stained cells. (F) Isx-9 significantly increased the number of Ki67-NeuN cells. Data are mean ± SD. *p<0.05 vs. control group.

Next, we asked whether Isx-9 could enhance neuronal differentiation. Immunostaining demonstrated that NSPCs treated with Isx-9 (6.25–50 uM for 5 days) showed an increase in mature neuronal markers neurofilament and NeuN, suggesting that Isx-9 can indeed promote NSPC differentiation (Fig 3A and 3B). As a positive control, BDNF treatment (10 ng/mL) also appeared to enhance NSPC differentiation into neurons (Fig 3A). However, maintaining cells in the proliferation medium continued to preserve NSPCs in an immature state (Fig 3A). Western blotting confirmed that 5-day Isx-9 treatment increased the protein levels of neurofilament and NeuN in NSPC cultures (Fig 3C and 3D). To further extend these findings, additional experiments were performed at the responsive concentration of 25 uM Isx-9. Exposure of NSPCs to this pro-neurogenic level of Isx-9 appeared to significantly increase numbers of Ki67-NeuN double-labeled cells (Fig 3E and 3F).

### Effects of Isx-9 on OPCs

**Fig 4:**
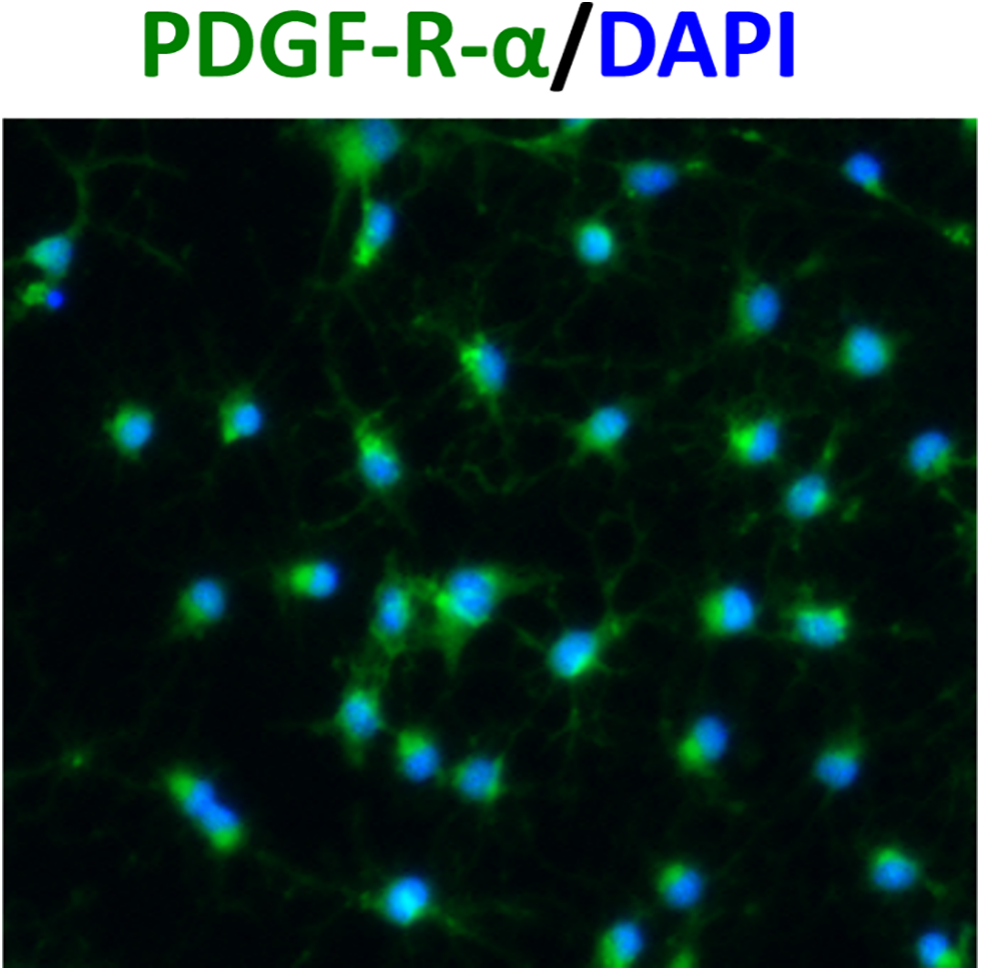
OPC cultures. Immunohistochmistry confirmed that our OPC cultures were positive for a OPC marker PDGF-R-α. Nuclei were stained by DAPI shown in blue.

**Fig 5:**
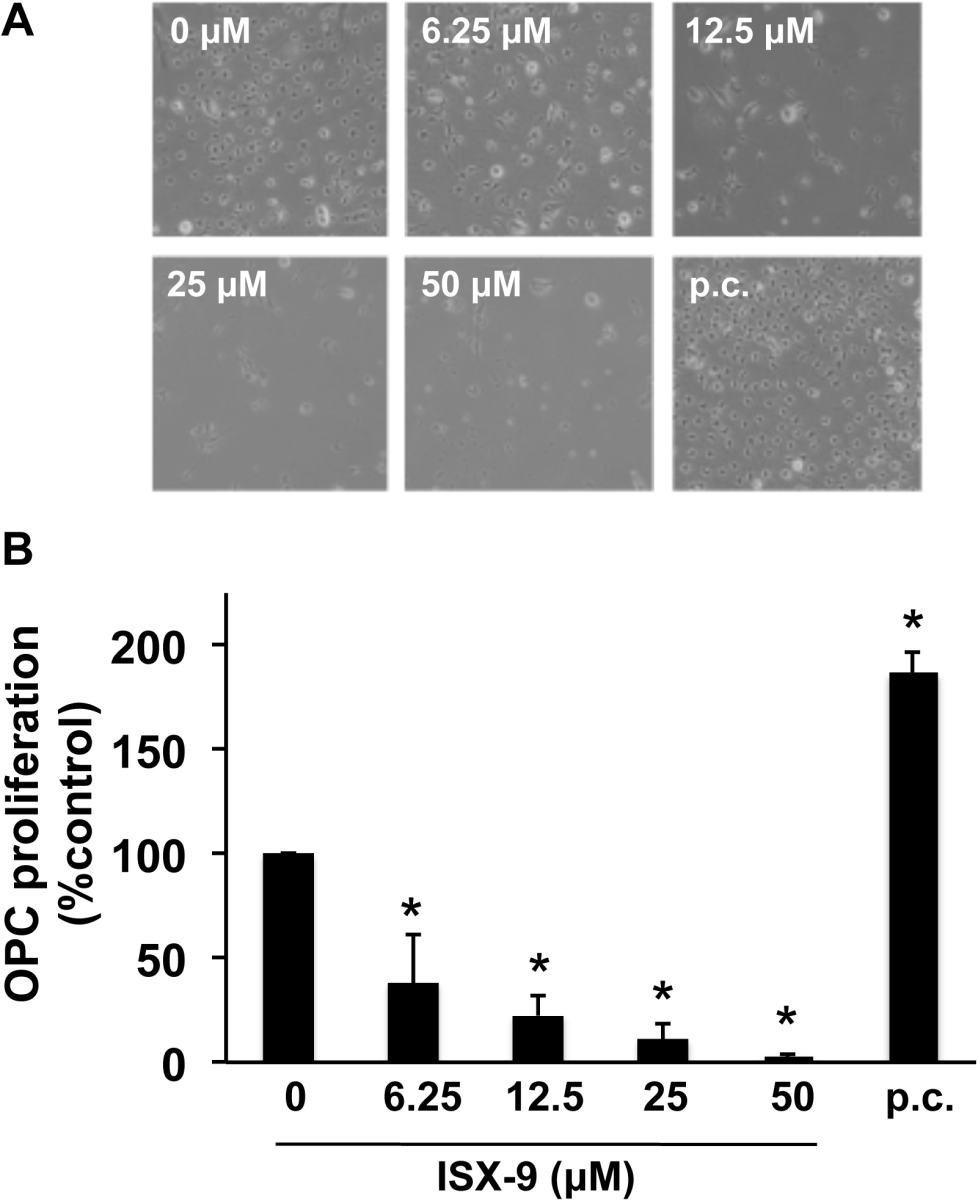
Isx-9 induced cell damage in OPCs. OPC cultures were treated with various concentrations of Isx-9, and two days later, their proliferation was evaluated by direct counting. Maintaining cells in the proliferative media was used as a positive control to proliferate OPCs. (A) Representative images of OPCs after Isx-9 treatment. (B) Isx-9 treatment decreased the number of OPCs in a concentration dependent manner. The positive control indeed increased the number of OPCs. Data are mean ± SD. *p<0.05 vs. control group. p.c. indicates positive control.

OPC cultures were positive for the standard OPC marker PDGF-R-α (Fig 4). Maintaining OPCs in their original proliferation medium (containing 10 ng/mL FGF-2 and 10 ng/mL PDGF) did indeed increase proliferation (Fig 5A and 5B), suggesting that our in vitro OPC culture system was viable. However, administration of Isx-9 (6.25–50 uM for 2 days) resulted in significant decrease in OPC numbers in a concentration-dependent manner (Fig 5A and 5B). Isx-9 appeared to be cytotoxic to OPCs in culture.

### Effects of Isx-9 on EPCs

**Fig 6:**
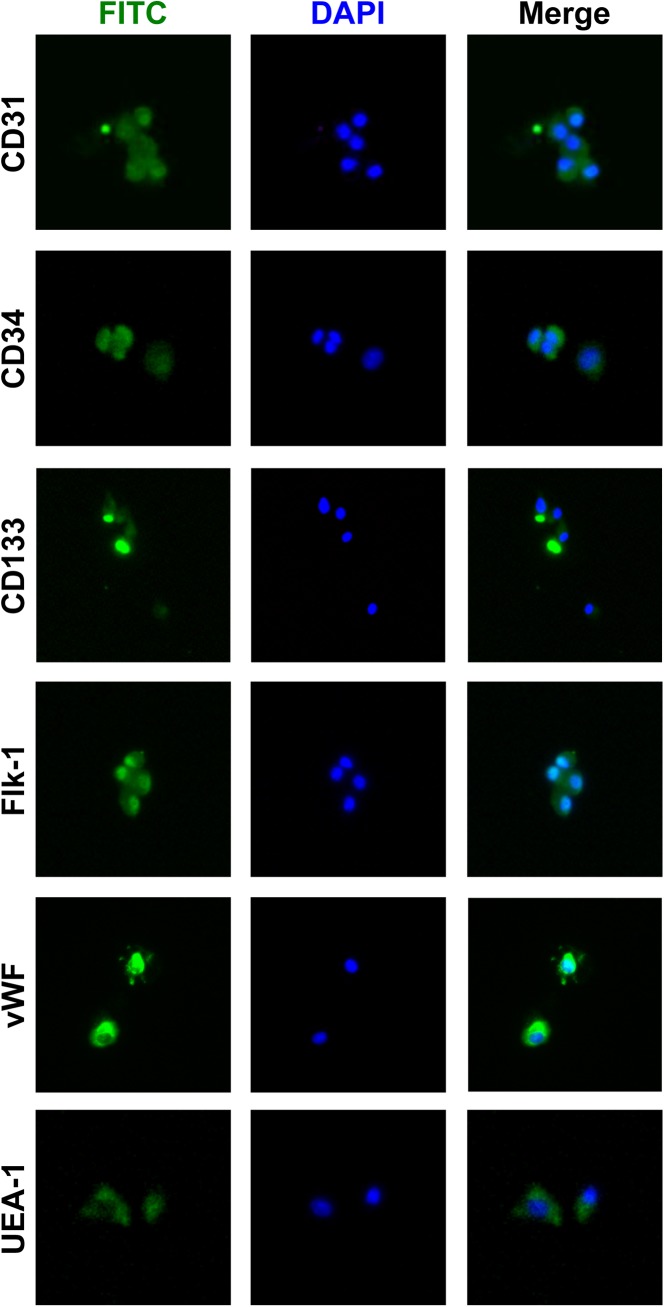
Early EPC cultures. Immunohistochmistry showed that our early EPC cultures were positive for CD31, CD34, CD133, Flk-1/VEGFR2, vWF, and UFA-1. Nuclei were stained by DAPI shown in blue.

**Fig 7:**
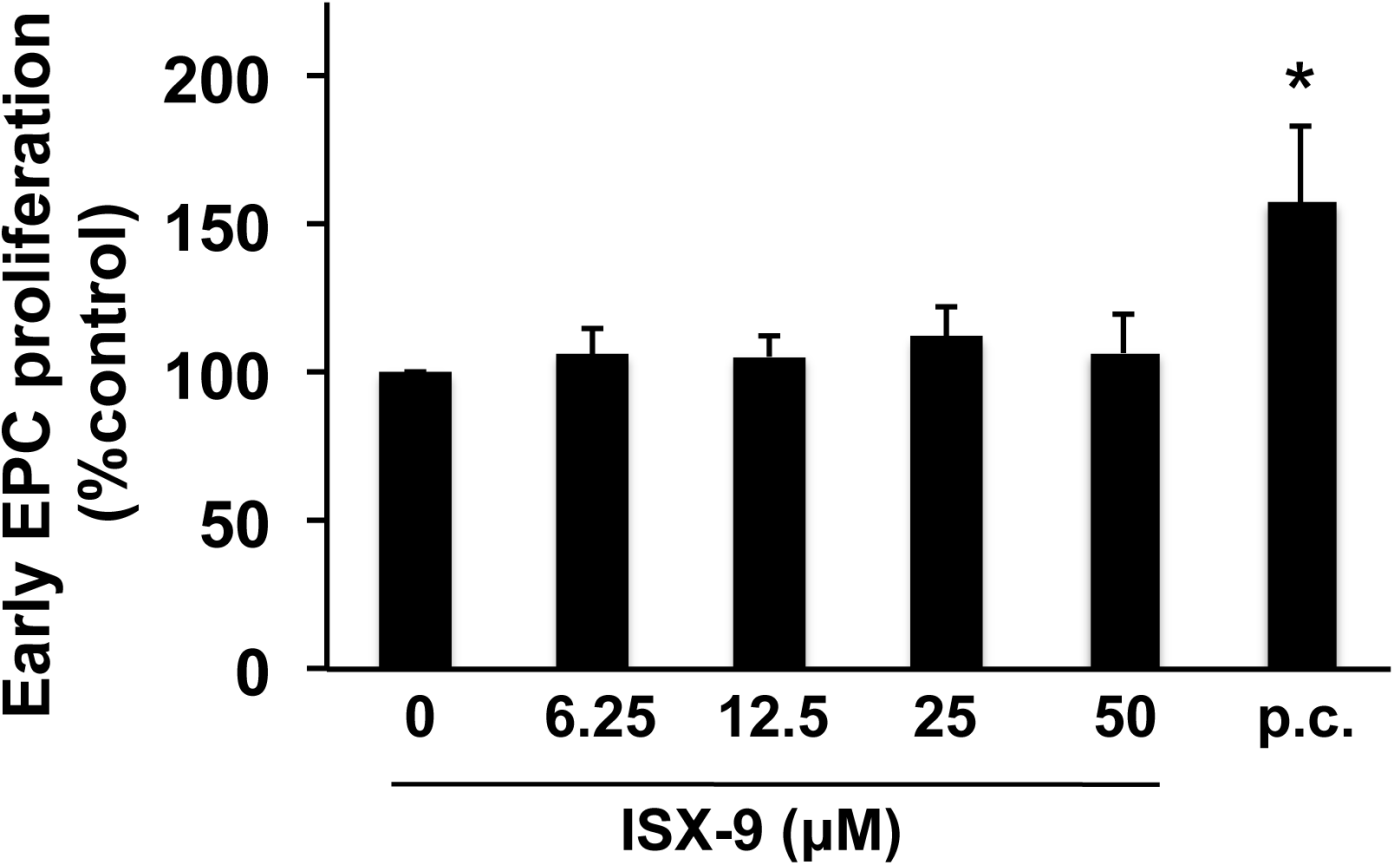
Isx-9 did not affect cell proliferation in early EPCs. Early EPC cultures were treated with various concentrations of Isx-9, and two days later, their proliferation was evaluated by direct counting. Maintaining cells in the proliferative media was used as a positive control to proliferate early EPCs. Isx-9 treatment did not change the cell number of early EPCs. The positive control indeed increased the number of early EPCs. Data are mean ± SD. *p<0.05 vs. control group. p.c. indicates positive control.

Standard early stage EPCs (5–6 days) expressed an array of markers expected for these cells, i.e. lectin-UFA1, Flk-1, vWF, CD31, CD34, and CD133 (Fig 6). Maintaining early stage EPCs in their original proliferation medium containing diverse trophic factors did indeed proliferate the cells further, demonstrating that this EPC system was viable (Fig 7). However, exposure of early stage EPCs to Isx-9 (6.25–50 μM for 2 days) had no detectable effects on cell proliferation (Fig 7).

**Fig 8:**
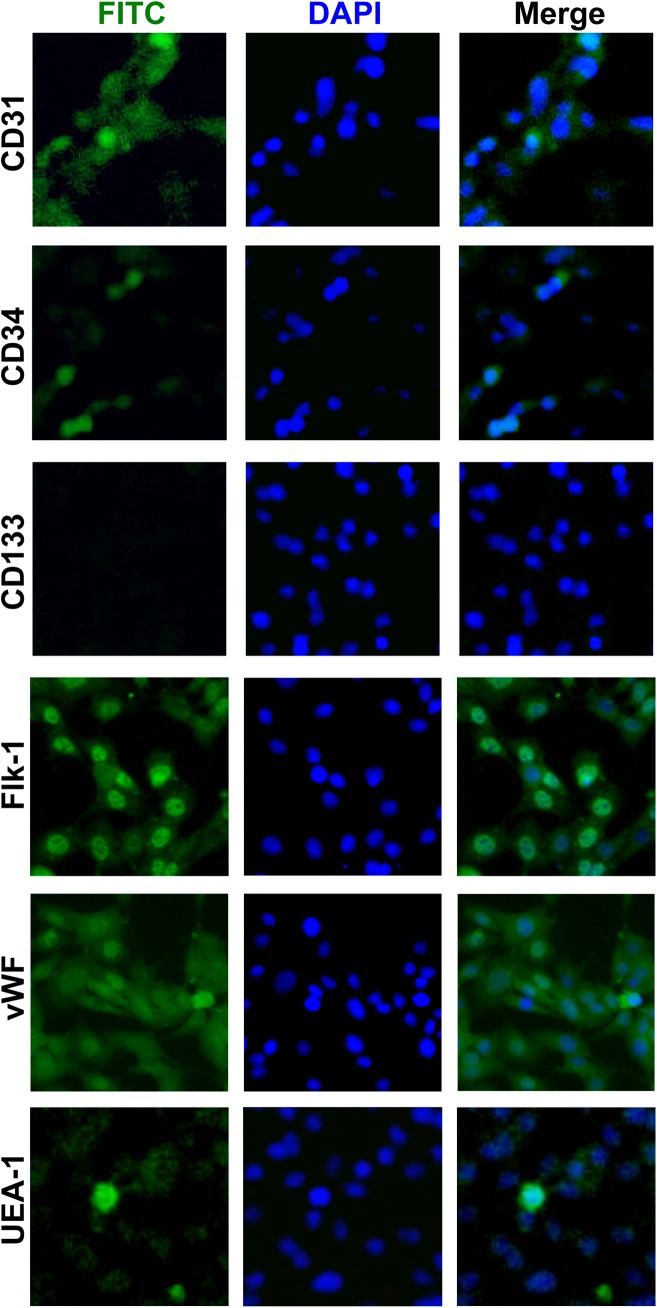
Outgrowh EPC cultures. Immunohistochmistry showed that our outgrowth EPC cultures were positive for CD31, CD34, Flk-1/VEGFR2, vWF, and UFA-1, but negative for CD133. Nuclei were stained by DAPI shown in blue.

**Fig 9:**
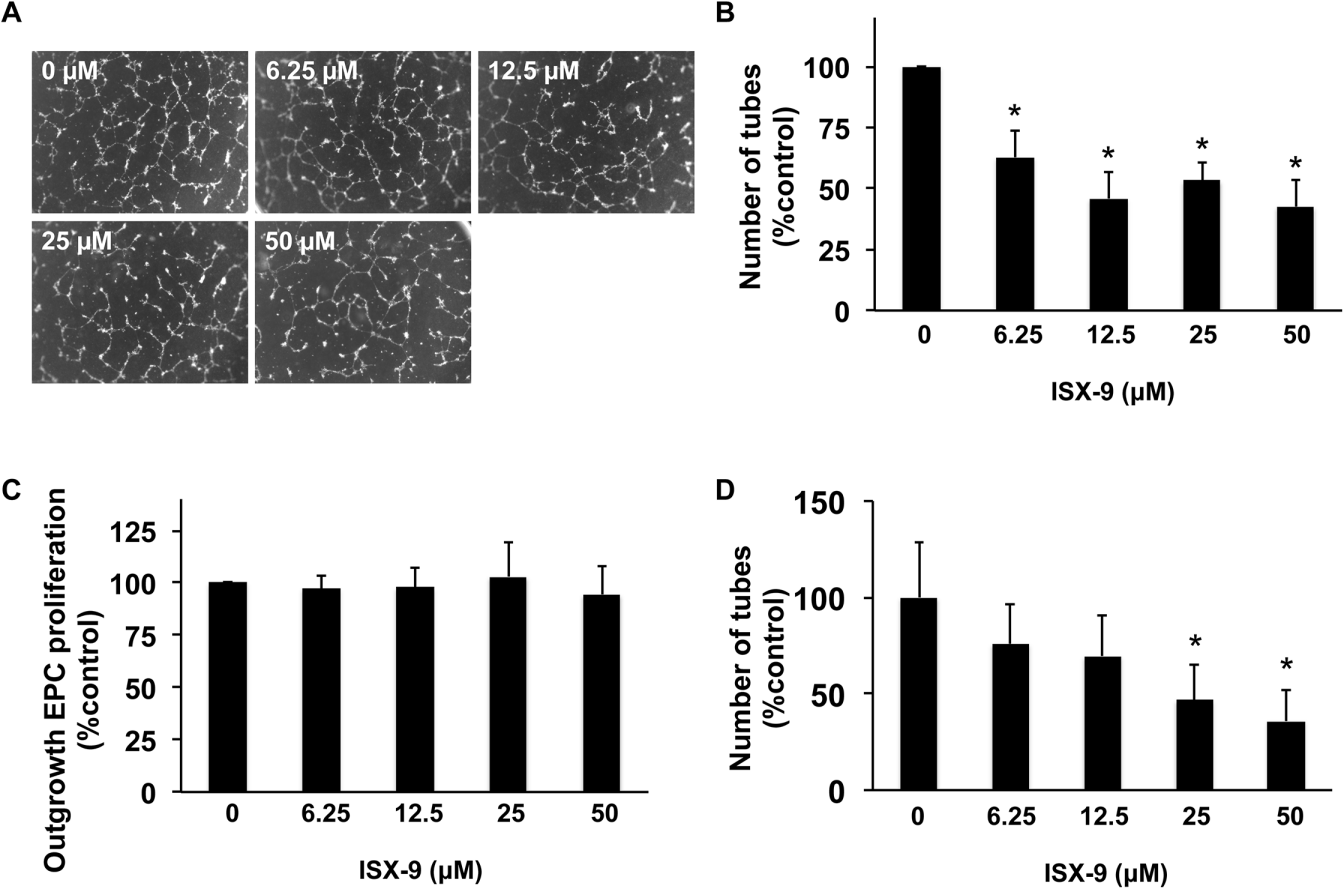
Isx-9 decreased tube formation in outgrowth EPCs. Outgrowth EPC cultures were treated with various concentrations of Isx-9, and one day later, their tube formation and proliferation were evaluated. (A) Representative images of outgrowth EPCs on the Matrigel plates after Isx-9 treatment. (B) Isx-9 treatment decreased the tube formation in a concentration dependent manner. (C) However, Isx-9 did not change the cell number of outgrowth EPCs. (D) Similar effects were observed in RBE.4 rat brain endothelial cells, where Isx-9 significant suppressed tube formation on the Matrigel assay. Data are mean ± SD. *p<0.05 vs. control group.

Late stage EPCs were obtained by growing out early EPCs for 1 to 1.5 months. These outgrowth EPCs expressed lectin-UFA1, Flk-1, vWF, CD31, and CD34, but as expected, CD133 expression was decreased (Fig 8). Outgrowth EPCs are known to possess endothelial properties. Therefore, we added Isx-9 to these cells and examined their angiogenic response in a Matrigel tube formation assay. Isx-9 treatment (6.25–50 μM for 1 day) significantly suppressed the number of vessel-like structures in a concentration dependent manner (Fig 9A and 9B) without changing cell survival/number (Fig 9C). As a positive control, proliferation media increased the number of complete ring structures, indicating that our in vitro angiogenesis assay was viable (data not shown). To confirm these potential anti-angiogenic effects of Isx-9, matrigel tube formation experiments were repeated with RBE.4 rat brain endothelial cells. Consistent with the late stage EPC findings, Isx-9 also significantly suppressed tube formation in mature rat brain endothelial cells (Fig 9D).

## Discussion

Boosting endogenous neurogenesis may be a promising therapeutic approach for diverse neurological diseases such as stroke, brain trauma and neurodegeneration [1–3]. In this regard, Isx-9 may represent a interesting initial approach since it has been demonstrated to enhance adult neurogenesis [6,7]. In this study, we tested the effects of Isx-9 on the three major progenitor cell types in brain, and demonstrated that Isx-9 (i) promotes the differentiation from NSPCs into neurons, (ii) causes cell damage in OPCs, and (iii) decreases angiogenic tube formation in late-stage EPCs. These findings suggest that mechanisms and pathways that underlie cell proliferation and differentiation may differ amongst various progenitor cell types. Development of pro-neurogenesis compounds for CNS recovery may require careful consideration of differential effects in multiple cell types.

In almost every CNS injury or disorder, loss of neurons is likely to underlie much of the disease phenotype. Hence, finding ways to replace lost neurons may be a logical approach. To achieve this purpose, stem cells have been used but many clinical trials using them have failed and these negative results led us to think about more strict steps toward cell therapy, especially for stroke [18]. Another way to boost endogenous neurogenesis is to develop chemical drugs and recent reports identify Isx-9 as a potential candidate [6,7]. Isx-9 belongs to a family of 3,5-disubstituted isoxazoles that were found in a screening effort to discover compounds that can convert neural precursors into mature neurons [6,7]. The mechanisms of Isx-9 are complex but are thought to involve calcium accumulation, activation of CaMKII, and modulation of HDAC pathways that trigger MEF2-related transcriptional signals for promoting neural differentiation. Beyond its original effects on NSPCs, however, it should be useful to also ask how Isx-9 may affect other types of precursor cells. Neurogenesis does not occur in isolation and crosstalk between multiple cell types is known to play key regulatory roles. For example, endothelium actively supports neurogenesis in the neurovascular niche by secreting a wide array of trophic factors [10]. In the context of disease, there also exists a causal association of regenerating neurons with the angiogenic vascular bed as damaged brain tissue attempts to repair itself [19,20]. Some of these neurovascular pathways may recruit circulating CD34+ EPCs [21] that may further contribute to vascular regeneration and neurovascular recovery [12,22,23]. The importance of EPCs and angiogenesis on neurogenic responses is also supported by the finding that systemic administration of human cord blood-derived CD34+ cells can enhance neurogenesis via upregulated angiogenic responses in a mouse model of cerebral ischemia [22]. Analogous mechanisms of cell-cell signaling may also exist in white matter. OPCs are precursor cells for myelin-forming mature oligodendrocytes that support axonal function in neurons. After white matter injury, OPCs become activated as part of an endogenous remodeling response [24]. Functional recovery cannot occur without proper reconnections in damaged or diseased white matter. Ultimately, any search for pro-neurogenic compounds must also take into account the parallel effects in progenitor cells from vascular and white matter compartments.

Taken together, our initial findings here suggest that Isx-9 may positively promote neurogenesis but negatively affect OPCs and EPCs. However, there are several important caveats to keep in mind. First, these results represent initial observations of cell-specific effects of Isx-9. Further studies are required to understand the differential mechanisms that lead to different outcomes in NSPCs, OPCs and EPCs. Second, the potential cytotoxicity in OPCs may be an important issue for further therapeutic development. Unlike NSPCs, OPCs may express functional calcium channels even at relatively immature stages. Is it possible that the known effects of Isx-9 on intracellular calcium accumulation might prove too much to handle for OPCs? More detailed studies into the underlying Isx-9-induced cell death mechanisms are warranted. Third, Isx-9 possesses HDAC inhibitory property [7], and HDAC inhibition was reported to enhance gene expressions of neuronal lineage but disturb the differentiation of OPCs into mature oligodendrocyte [25]. Therefore, future studies into HDAC signaling in NSPCs versus OPCs and EPC may be useful. Fourth, our experiments were performed in a single cell-type culture fashion. Cell-cell interactions are known to significantly influence cell homeostasis. Multi-cell culture systems should be explored in future studies. Fifth, our primary cell cultures were all derived from rodents (NSPCs and OPCs were from rats, and EPCs were from mice). In terms of drug discovery, testing the efficacy of Isx-9 or other analogs and candidates may benefit from studies performed in human cells. Finally and most importantly, this is only a proof-of-concept study. Whether these rat and mouse cell culture observations also apply to human cells or whether these phenomenon occur in vivo remains to be carefully investigated. Predicting inter-species responses is not easy but at least, our data here appear consistent in rats and mice. In terms of translation from cell culture to in vivo dosing, there is also no guaranteed approach. In our precursor/progenitor cell systems, we used Isx-9 levels in the range of 12.5 to 25 uM. Previous studies have suggested that intraperitoneal dosing in the range of 20 mg/kg over 1–2 weeks may augment neurogenesis in mouse brains in vivo [6]. In the end, although our data are consistent with previous reports on neurogenesis, no final conclusions can be made as to whether Isx-9 may have negative effects on white matter or brain vascular regulation in humans in vivo. However, the potentially negative effects in OPCs and EPCs suggest that further investigations are warranted.

In this proof-of-concept study, the pro-neurogenesis compound Isx-9 promoted proliferation and differentiation in primary rat NSPCs, but did not appear to promote proliferation in primary rat OPCs or primary mouse EPCs. Instead, Isx-9 may potentially increase cell death in OPCs and suppress in vitro angiogenesis in late-outgrowth EPCs. These findings suggest that mechanisms and targets for promoting endogenous stem or precursor cell recovery may differ amongst different neural, glial and vascular cell populations.
